# Is Bacterial Persistence a Social Trait?

**DOI:** 10.1371/journal.pone.0000752

**Published:** 2007-08-15

**Authors:** Andy Gardner, Stuart A. West, Ashleigh S. Griffin

**Affiliations:** 1 St John's College, Oxford University, Oxford, United Kingdom; 2 Institute of Evolutionary Biology, University of Edinburgh, Edinburgh, United Kingdom; University of California at Berkeley, United States of America

## Abstract

The ability of bacteria to evolve resistance to antibiotics has been much reported in recent years. It is less well-known that within populations of bacteria there are cells which are resistant due to a non-inherited phenotypic switch to a slow-growing state. Although such ‘persister’ cells are receiving increasing attention, the evolutionary forces involved have been relatively ignored. Persistence has a direct benefit to cells because it allows survival during catastrophes–a form of bet-hedging. However, persistence can also provide an indirect benefit to other individuals, because the reduced growth rate can reduce competition for limiting resources. This raises the possibility that persistence is a social trait, which can be influenced by kin selection. We develop a theoretical model to investigate the social consequences of persistence. We predict that selection for persistence is increased when: (a) cells are related (e.g. a single, clonal lineage); and (b) resources are scarce. Our model allows us to predict how the level of persistence should vary with time, across populations, in response to intervention strategies and the level of competition. More generally, our results clarify the links between persistence and other bet-hedging or social behaviours.

## Introduction

Within a population of bacteria there exists a subgroup of cells that do not grow at the normal rate but exists in a quiescent, non-growing or slow-growing state. These cells are sometimes called persister cells [1], because they are able to persist in the face of catastrophic events such as antibiotic treatment [2]–[17]. In the case of antibiotic treatment, persister cells are able to survive because an important action of antibiotics relies on disrupting translation of the mRNA code to polypeptide chains, and this process does not occur in non-growing cells. A key aspect of persister cells is that their resistance to antibiotic treatment is not genetically determined. Consequently, following antibiotic treatment, persisters give rise to new populations that have the same vulnerability to antibiotic treatment as the ancestral population [9]. The resistance of persister cells is therefore determined phenotypically, with cells switching between the alternative phenotypic states of persistence and normal growth [7], [11].

Although much effort has gone into understanding the mechanistics of persistence, little attention has been given to the evolutionary forces that explain why persistence should be favoured [9], [18]. The prevalent view within the microbial literature is that this behaviour can explained by benefits accruing at the level of the population, since persister cells represent an insurance policy that permits population survival in the event of catastrophe [2]–[3], [10], [13]–[15]. However, the idea that traits are favoured because they benefit the population was generally rejected in the evolutionary literature by a large body of theoretical and empirical work instigated in the 1960s [19]. Instead, it is necessary to consider the costs and benefits of a trait, both for the individual that performs it, and for those that they interact with. Persistence has a direct benefit to cells because it allows survival during catastrophes. In this respect it can be compared with other bet-hedging strategies such as seed dormancy and insect diapause [20].

However, persistence can also provide an indirect benefit to other individuals, because the reduced growth rate can reduce competition for limiting resources. This raises the problem that cells that allocate more time to the persister state could be out-competed by cells which allocate less time to the persister state, and instead invest more heavily in growth. We develop a theoretical model that makes explicit the direct (selfish) and indirect (social) fitness consequences of persistence. This allows us to predict how the evolutionary stable level of persisters depends upon population demographic parameters such as intensity of resource competition, the frequency of catastrophes such as antibiotic treatment, and the genetic structure (heterogeneity) of populations. Our model allows us to investigate the evolutionary conflict (tension) between the interests of the individual, and that of the group or population. This clarifies links to other microbial social traits, as well as organisms that are more often studied from a social perspective such as ants, and other social animals.

## Analysis

**Figure 1:**
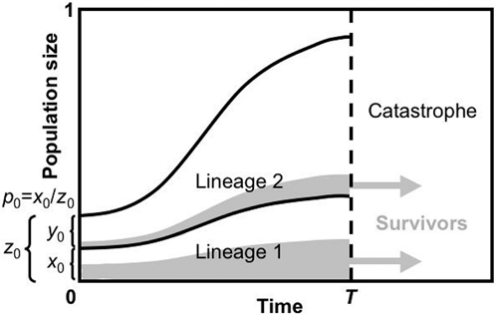
An illustration of the basic model, defining the three dimensionless model parameters (*T*, *z*_0_ and *p*_0_). Two lineages compete for resources during the growth interval before catastrophe occurs at time *T*. The initial size of the focal lineage is *x*_0_, expressed as a proportion of the total carrying capacity. The initial size of the total population is *z*_0_ = *x*_0_+*y*_0_, where *y*_0_ is the initial size of the competitor lineage. The initial frequency of the focal lineage is *p*_0_ = *x*_0_/*z*_0_. Persister cells are represented by the shaded areas, and non-persister cells are unshaded. Upon the catastrophe occurring, all persister cells survive and all non-persister cells are destroyed.

We consider a large metapopulation of bacteria structured into many separate patches (representing, for example, host individuals), and we investigate reproductive and survival success within a focal patch. Bacterial growth in the context of resource competition is captured by the standard logistic growth model, where the local population moves from exponential growth phase towards stationary phase as it increases in size and resources are exhausted (Figure 1). The model notation is summarized in Table 1. At any time *t* the local population size *z_t_* is expressed as a proportion of the patch carrying capacity, and the initial size of the population (*z*_0_, at time *t* = 0) provides the first parameter for our model. The local population comprises two lineages, X and Y, and the size of the lineages are denoted *x_t_* and *y_t_*, such that *x_t_*+*y_t_* = *z_t_*. It is helpful to denote the proportion of the population that belongs to lineage X as *p_t_* = *x_t_*/*z_t_*, and the initial proportion (*p*_0_, at time *t* = 0) provides the second parameter of the model.

**Table 1:** A summary of model notation.

*Symbol*	*Definition*
*T*	Time
X	Focal lineage
Y	Competitor lineage
*x_t_*	Size of the focal lineage in the local population at time t
*y_t_*	Size of the competitor lineage in the local population at time t
*z_t_*	Local population size at time t
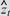	Local population size at time t in the neutral case
*z*_0_	Local population size at time t = 0
*ζ_t_*	Effective local population size at time t (extended model)
*p_t_*	Frequency of the focal lineage in the local population at time t
*p*_0_	Frequency of the focal lineage in the local population at time t = 0
*℘_t_*	Effective frequency of the focal lineage at time t (extended model)
π	Wildtype persister allocation strategy, adopted by competitor lineage
π+δπ	Variant persister allocation strategy, adopted by focal lineage
π*	Evolutionarily stable persister allocation strategy
*T*	Waiting time until catastrophe
*T̅*	Average waiting time until catastrophe (extended model)
*a*	Relative competitive strain of persisters (extended model)
*g*	Relative growth rate of persisters (extended model)
*s*	Relative nonpersister survival through catastrophes (extended model)
*P_t_*	Number of focal-lineage persister cells at time t
*w*	Darwinian fitness of focal lineage

Persister allocation (π) is defined as the proportion of time spent in the persister state, and for simplicity we assume that this remains fixed throughout the growth period (but see discussion). The two lineages differ slightly in their persistence behaviour: lineage X allocates a proportion π+δπ of its time in the persister state, whereas lineage Y allocates a proportion π; we assume that δπ is a vanishingly small quantity. Genetic variation has been shown to exist between lineages in their allocation to the persister state [7], [11]. We assume that cells in the persister state exhibit zero growth, and cells in the non-persister state grow at a baseline rate. Because the units of time are arbitrary, we define our basic time unit such that the instantaneous baseline growth rate is unity. Finally, we assume that the growth period is interrupted by a catastrophic event, such as antibiotic treatment, where all persister cells survive while all non-persister cells are destroyed. Our model can be extended to more complicated situations such as less extreme differences in growth and survival rates (see Appendix S1), but this does not alter the qualitative nature of our results.

We equate the absolute number of persister cells achieved by a lineage at the moment of catastrophe to the Darwinian fitness of that lineage. Initially, we consider that the timing of the catastrophe is regular and occurs after *T* time units; this provides the third parameter for our model. In Appendix S1 we extend the model to allow catastrophes to strike at random times, with average waiting time until catastrophe given by the parameter *T̅*, which recovers the same qualitative results.

**Figure 2:**
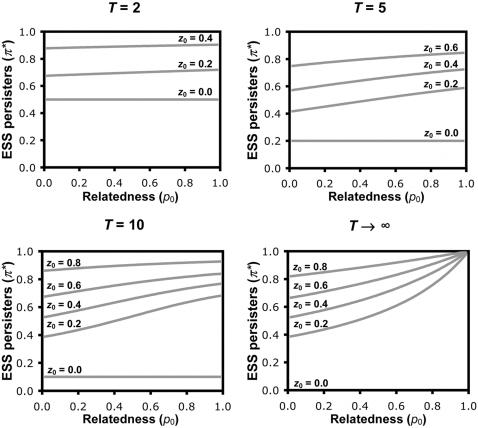
The evolution of persister function, assuming fixed time until catastrophe. Numerical solutions for the ESS persister allocation are given for a range of parameter values. The ESS allocation to persister function (π*) decreases as the growth time (*T*) before catastrophe increases, and the ESS increases with increasing resource competition (*z*_0_) and genetical relatedness (*p*_0_). Note that *T*→∞ does not imply infinite growth, but rather that the catastrophe occurs after resources are exhausted and growth has ceased. Also, some proportion of persisters is always favoured (i.e. π*>0), but the quantity predicted may be vanishingly small and hence appear to be zero in the figure.

**Figure 3 pone-0000752-g003:**
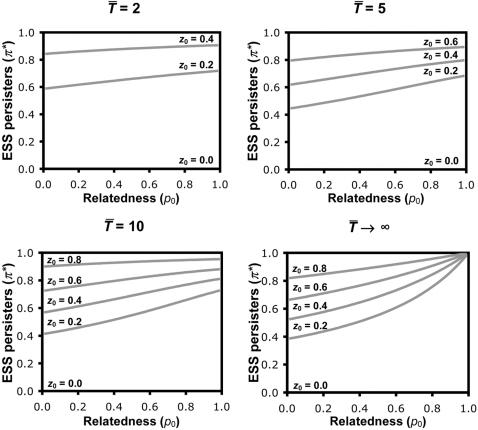
The evolution of persister function, with random waiting time until catastrophe. Numerical results for the stochastic version of the model in which the probability of catastrophe occurring is at any time is constant through time, with average waiting time *T̅*. The ESS allocation to persister function (π*) decreases as the average growth time (*T̅*) before catastrophe increases, and the ESS increases with increasing resource competition (*z*_0_) and genetical relatedness (*p*_0_). Note that *T̅*→∞ does not imply infinite growth, but rather that the catastrophe (almost always) occurs after resources are exhausted and growth has ceased. Also, some proportion of persisters is always favoured (i.e. π*>0), but the quantity predicted may be vanishingly small and hence appear to be zero in the figure.

**Figure 4:**
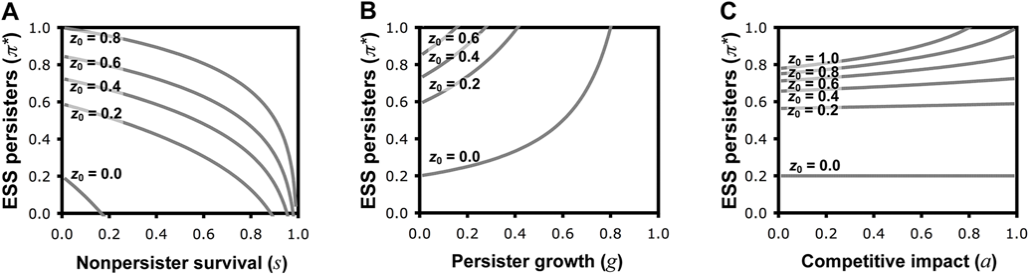
The evolution of persister function, with less extreme differences in persister and nonpersister survival and growth, and differences in efficiency of resource use. (A) ESS persister allocation (π*) is a decreasing function of the relative survival (*s*) of nonpersister cells. We assume: *p*_0_ = 1, *T* = 5, *g* = 0, *a* = 1 and a range of *z*_0_. (B) ESS persister allocation (π*) is an increasing function of the relative growth rate (*g*) of persister cells. We assume: *p*_0_ = 1, *T* = 5, *s* = 0, *a* = 1 and a range of *z*_0_. (C) ESS persister allocation (π*) may depend on the relative competitive strain on resources imposed by persister cells (*a*), but this is negligible when they appear only infrequently in bacterial populations (low π*). We assume: *p*_0_ = 1, *T* = 5, *s* = 0, *g* = 0 and a range of *z*_0_.

From an evolutionary perspective, the key problem with persistence is the trade-off between survival and growth. Persistence is beneficial because it allows survival through catastrophic events, but is costly because it leads to lower growth rate. Our aim is to determine the evolutionarily stable strategy (ESS; [21]) for proportional allocation to persister function (π*) for given situations, i.e. as a function of model parameters (*z*_0_, *p*_0_ and *T*). This is the strategy that is the best response to itself; in other words, when all members of the metapopulation adopt the ESS, each individual maximizes its fitness by adopting the ESS, and those that employ a variant strategy do not achieve higher fitness. Consequently, when the metapopulation is not at an ESS, rare variants adopting different persister strategies can invade, and when the majority of individuals adopt the ESS, no other persister strategy can invade from rarity. In Appendix S1, we calculate how persister strategies relate to growth and survival through catastrophe events, and hence Darwinian fitness, and arrive at an implicit solution for the ESS:
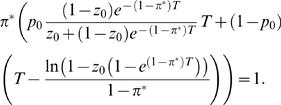
(1)This can be solved explicitly for numerical parameter values (Figure 2). The same procedure can be used to derive numerical solutions for the extended models incorporating random waiting times until catastrophe (Appendix S1, and Figure 3), less extreme differences between persister and nonpersister cells in their growth or survival rates, and differences in the competitive strain that they exert upon the population's resources (Appendix S1, and Figure 4).

## Discussion

### Catastrophes and Resource Competition

Our basic model allows us to investigate the influence of three factors on the selective advantage of persistence. First, we can vary the frequency with which population catastrophes such as antibiotic treatment occur. In our model, catastrophe occurs after *T* time units, with lower values of *T* corresponding to catastrophes happening more frequently. As catastrophes become more frequent (lower *T*), the ESS persister allocation increases (Figure 2), as was also predicted in an earlier study [11]. This is because there is less time to grow, and so the growth cost of persistence is reduced and selection for improved survival becomes relatively more important. Extending the model, so that catastrophe strikes at random, we recover the same qualitative results (Figure 3). Further extensions show that improving the chance of survival of nonpersister cells through catastrophes reduces the ESS allocation to persister function (Figure 4A), and that allowing persisters to exhibit growth increases the ESS persister allocation (Figure 4B), highlighting that the benefit of persistence is a relatively-higher survival through catastrophes and the cost of persistence is a relatively-lower rate of growth. These results emphasise the relationship between persistence and other bet-hedging strategies that provide a direct (personal) fitness benefit, such as seeds entering dormancy in case of bad years [20].

A complication with persistence, compared with other bet-hedging strategies, is that it can also have significant social consequences. Behaviours (or other traits) are social if they have fitness consequences, either positive or negative, for both the individual that performs the behaviour and another individual or individuals [19]. The social nature of persistence is illustrated by considering a second factor: resource competition, as influenced by initial population size, *z*_0_. Low values of *z*_0_ (*z*_0_≈0) correspond to small populations initially undergoing essentially exponential growth with little competition for resources. As *z*_0_ is increased, the population is larger, and closer to stationary phase, and hence there is greater competition for resources. We find that when there are higher levels of resource competition, with higher values of *z*_0_, greater levels of persistence are favoured (Figures 2–4). Higher competition for resources means reduced potential for growth, which reduces the direct growth cost of persister function, and increases the indirect benefit of reducing competition for relatives. This result emphasises the indirect social consequences of persistence, and that it will be influenced by the relatedness of interacting individuals (kin selection). Specifically, when there is competition for resources, persistence provides a benefit for both the individual and its neighbours. Behaviours which benefit another individual are termed cooperative, and cooperative behaviours which also benefit the individual that performs the behaviour are termed mutually beneficial [19]. A further social benefit arises if persistence results in more efficient use of resources, so that a higher carrying capacity could be achieved when the population allocates more to persister function. While this would appear to favour increased investment into persistence, the secondary effect of reducing the intensity of resource competition, which directly disfavours persistence, means that there is no simple relation between their efficiency and the ESS. However, the impact of relative persister efficiency is negligible when they are present at a low frequency (Figure 4C), which is generally the case in bacterial populations.

Our result that higher competition for resources favours greater allocation to persistence, is in the opposite direction to the general result from numerous other models, where competition for local resources selects for lower levels of cooperation [22]. The difference here is that other cooperative behaviours, such as the production of public goods in bacteria, or cooperative breeding in vertebrates, generally increase population growth, and hence can increase competition for resources between relatives (indirect cost; [22]–[23]. In contrast, persistence reduces population growth, and hence decreases competition for resources between relatives (indirect benefit). Consequently, persistence is not exactly analogous to apparently similar cooperative behaviours, such as the production of reserve queens in stingless bees [24].

### Persisters and the Growth Cycle

Our model assumes that persister allocation is fixed throughout the growth of a lineage. However, we can infer from the above prediction that the proportion of cells in the persistence state should increase during the growth cycle of a bacteria population. Earlier in the growth cycle, when there is an abundance of resources and essentially exponential growth, corresponds to a low value of *z*_0_, and hence we predict a relatively low proportion of persisters. This is because at this stage there is a greater gain to be made from allocating resources to short term growth. In contrast, later in the growth cycle, when there is greater competition for resources during the approach towards stationary phase, corresponds to a high value of *z*_0_, and hence we predict a greater proportion of persisters. This is because when growth rates are slow anyway, there is a lower cost to entering the persistence state, and a greater indirect benefit (see above).

Our prediction that there should be a lower proportion of persister cells in the exponential phase is supported by data from both *in vivo* and *in vitro* studies on growth rate and the efficacy of antibiotics [16]. Keren et al. [8] found that tolerance to antibiotic treatment due to persister cells increased dramatically in mid-exponential phase, i.e. upon approaching stationary phase. Our model predicts that in populations with lower growth rates, a higher proportion of cells will be in the persister state. Consequently, we can predict that antibiotic treatment should be less successful in populations with lower growth rates. There is considerable support for this prediction [16]. It has been known since the 1940s that the rate at which bacteria are killed by penicillin is directly proportional to the rate of growth *in vitro* [25] and *in vivo* [26]. More recently, Cozens et al. [27] compared the efficacy of 23 different antibiotics in treating infections of five different species of bacteria, using a chemostat emulating *in vivo* growing conditions. They found that bacteriocidal activity decreased proportionately with slower growth rate in all but a few cases.

### Relatedness

Third, we consider consequences of varying genetic relatedness (or diversity; [28]–[31]) within the population. This is done by varying the population frequency of the focal bacterial lineage (*p*_0_). Since the evolutionarily stable strategy (π*) is assessed by examining the invasion success of a lineage that adopts a variant persister allocation strategy and is introduced into the metapopulation at a vanishingly small frequency, then the local frequency of the focal lineage (*p*_0_) is exactly equivalent to the kin selection coefficient of relatedness (*r*), which describes the average genetic similarity of patch-mates relative to the metapopulation as a whole. We found that higher relatedness (*p*_0_) selects for higher levels of persistence (Figures 2–3). One way to conceptualise this is that slow growth reduces competition between relatives by freeing up resources. This effect is analogous to that in evolutionary models that examine how exploitation host strategies influence parasite virulence [32].

The influence of genetic relatedness illustrates the conflict of interest between the individual cell, and the local group or population. When *p*_0_ = 1 the population is clonal, so there is no conflict between the interests of each individual and the population as a whole, and the ESS persister allocation (π*) predicted here represents the optimum for the population. However, reducing *p*_0_ results in a genetically heterogeneous population (lower relatedness), where there is scope for a selfish lineage to cheat by allocating more to growth, and thereby monopolise resources. Although such a selfish strain would allocate proportionally less to persister function, its enhanced growth could lead to a greater number of persister cells at the time of catastrophe. This conflict of interests leads to selection favouring a persister strategy π* that is lower than the population optimum (Figures 2–3). Analogous conflicts of interest can be found in a range of social organisms from slime-moulds to ants to humans [33]–[34].

Our results demonstrate that persistence can be a social trait, but that the relative importance of social factors will depend upon population demography. Traits are social if they have fitness consequences for both the individual that performs the behaviour and another individual or individuals [19], [29]. Persistence can provide a direct fitness benefit and so can be selected for by purely selfish reasons, as shown by the fact that it can be favoured even when relatedness is zero (*p*_0_ = 0; Figures 2–3). However, except in the extreme case of exponential growth (*z*_0_ ≈ 0), persistence also provides a benefit to other individuals in the population, by decreasing competition. It is this effect on competition that can make persistence a social trait, which can be influenced by kin selection. More specifically, it is a mutually-beneficial cooperative trait that will be favoured at higher levels when the relatedness between interacting cells is higher. A trait is cooperative if it provides a benefit to another individual or individuals [19], and hence allows the possibility for indirect fitness benefits through helping relatives, as shown by how increased relatedness (higher *p*_0_) favours greater levels of persistence (Figures 2–3). The relative importance of the direct and indirect benefits of persistence will vary with the value of parameters such as *p*_0_ and *z*_0_. For example, when relatedness (*p*_0_) and competition for resources (*z*_0_) are low, the direct benefit of persistence will be the primary selective force, and indirect benefit to others will just be a byproduct. In contrast, as relatedness and competition for resources increase, a higher level of persistence is favoured due to its indirect benefits. A key point here is that we are not making any claim about the relative importance of the individual (direct) and social (indirect) fitness consequences of persistence–that will require empirical work. Our aim here is merely to show that persistence could potentially also have social fitness consequences (reducing competition), in addition to the well accepted individual benefit (surviving catastrophes).

Our results emphasise the similarities and differences between persistence and analogous bet-hedging traits such as seed dormancy [20], [35]–[36] or insect diapause [20], [37]–[38]. In all of these cases there can be a direct fitness benefit to the dormancy trait because it can allow survival during hard times. What sets persistence apart is that it can also have important indirect fitness consequences for relatives, through reducing competition for resources. While there are possibilities for behaviours such as insect diapause or seed dormancy to alter the potential for competition between relatives [39], any effects are likely to negligible in most real cases [35], [37]. In contrast, the extreme population (genetic) structure that arises due to fast clonal growth means that bacterial persistence could have considerable indirect effects.

### Extending the Model

We have used a simplified model to demonstrate that bacterial persistence can be a social trait and that increasing competition and relatedness will tend to favour more time spent in the persister state. These qualitative results are expected to be robust over a wide range of possible models. However, it would be extremely useful to extend this model in a number of directions, especially for quantitative application to specific species. We have assumed, for lack of reliable data, that there is negligible turnover of cells during stationary phase. Although such turnover is invisible at the ecological level, because population sizes remain at a fixed dynamic equilibrium, there is scope for natural selection as certain lineages outcompete others, and hence potentially important evolutionary implications [40]. This turnover should not alter the qualitative predictions of the present model, but may have a significant quantitative impact on the evolution of many bacterial traits. Also, we have assumed all patches are equivalent and all catastrophes are identical. More realistically, bacteria are expected to find themselves subjected to a range of different environmental conditions, and population disturbances can vary in their intensity and underlying cause–so while persisters may be refractory to antibiotic treatments, they could be susceptible to catastrophes of a different nature. These suggest interesting avenues for further exploration, but we expect the qualitative results of the present analysis to remain intact.

It is reasonable to suspect that certain characteristics of the model, including initial size and genetic heterogeneity of the bacterial populations at the beginning of the growth phase, might vary as a consequence of the allocation of cells to the persister state. We have taken an ‘open-model’ approach [41] that assumes that these characteristics can be adequately represented by fixed parameters that can be varied independently of each other. More generally, these could be evolving, co-dependent variables in their own right, and this could be shown by taking a ‘closed model’ approach [41] that allows such characteristics to emerge from concrete population processes such as the pattern of dispersal and the mutational input of novel genetic material. However, the closed-model approach requires restrictive assumptions that limit the generality of a model. The open-model approach sacrifices some realism for a more straightforward analysis and conceptually simple results that are more readily generalisable and easy to relate to other models. With this in mind, it is important to emphasise that the present analysis is intended to highlight the social evolutionary implications of bacterial persistence, which provides a new avenue of inquiry into this important topic, and not to make quantitative predictions for particular species.

### Conclusions

We have shown that persistence can be a social trait, and that it can be influenced by aspects of population structure that determine genetic homogeneity (relatedness) and resource availability (competition) within populations. Our model provides a complementary approach to previous work by Kussell et al. ([11]; see also [7]), which focused upon the importance of catastrophes and allowed successful quantitative predictions for a specific system. Their model assumed the special case where no social interactions occur, due to exponential growth (*z*_0_≈0) and only one genetic lineage per population (*p*_0_ = 1). Our model makes predictions that could be tested in either controlled laboratory experiments or clinical situations. Laboratory experiments would allow the key demographic parameters to be manipulated independently, whereas clinical intervention strategies are likely to influence multiple factors simultaneously. For example, more frequent antibiotic treatment could lead less genetically diverse populations (higher *p*_0_), or a higher proportion of growth under less resource competition (lower *z*_0_). More generally, our model emphasises that because of the selfish interest of individuals, bacteria will not necessarily be selected to behave in ways that optimise population survival. It will be extremely useful to apply similar evolutionary thinking to a range of microbial social traits that have previously been assumed to be optimising population survival [18], [42]–[45].

## Supporting Information

Appendix S1 Appendix.
(0.53 MB PDF)
