# Supplementary material for: Is Bacterial Persistence a Social Trait?
Source: PLoS One. 2007 Aug 15;2(8):e752. doi: 10.1371/journal.pone.0000752 (PMC1937027; doi:10.1371/journal.pone.0000752)
Supplement: Appendix S1 — Appendix. (0.53 MB PDF) [file pone.0000752.s001.pdf]

Appendix

Basic Model

We follow the growth dynamics of the two bacterial lineages within the focal patch, to determine the number of cells of the focal lineage that survive catastrophe. The growth of the two lineages, X and Y, is given by:

$$\frac{\mathrm{d}x}{\mathrm{d}t} = \left(1 - (\pi + \delta\pi)\right)x_t\left(1 - (x_t + y_t)\right), \text{ and} \tag{A.1}$$

$$\frac{\mathrm{d}y}{\mathrm{d}t} = \left(1 - \pi\right)y_t\left(1 - (x_t + y_t)\right). \tag{A.2}$$

The growth of the whole population is:

$$\frac{\mathrm{d}z}{\mathrm{d}t} = \left(1 - (\pi + p_t\delta\pi)\right)z_t\left(1 - z_t\right), \tag{A.3}$$

where $p_t = x_t/z_t$. From the quotient rule, the change in this frequency is given by:

$$\frac{\mathrm{d}p}{\mathrm{d}t} = \left(z_t\frac{\mathrm{d}x}{\mathrm{d}t} - x_t\frac{\mathrm{d}z}{\mathrm{d}t}\right) / z_t^2 = -p_t\left(1 - p_t\right)\left(1 - z_t\right)\delta\pi . \tag{A.4}$$

Noting that changes in frequency are vanishingly small in this nearly-neutral case, we can write $p_t = p_0 + O(\delta\pi)$ where p_0 is the starting frequency of the focal lineage. The differential equation (A.3) yields the general solution:

$$z_t = \frac{z_0}{z_0 + (1 - z_0)e^{-(1-\pi)t}}\left(1 - p_0\left(1 - \frac{z_0}{z_0 + (1 - z_0)e^{-(1-\pi)t}}\right)t\delta\pi\right) + O\left(\delta\pi^2\right), \tag{A.5}$$

where z_0 is the initial population size. The differential equation (A.4) yields the general solution:

$$p_t = p_0 - p_0(1 - p_0)\left(t - \ln\left(1 - \frac{z_0(1 - e^{(1-\pi)t})}{1 - \pi}\right)\right)\delta\pi + O\left(\delta\pi^2\right). \tag{A.6}$$

At time t the number of cells in the focal lineage is $x_t = p_t\, z_t$, and the number of persister cells belonging to the focal lineage is $P_t = (\pi+\delta\pi)x_t$, which is given by:

$$P_t = \pi p_0 \hat{z}_t + p_0 \hat{z}_t \left(1 - \pi\left(p_0\left(1-\hat{z}_t\right)t + \left(1-p_0\right)\left(t - \ln\left(1 - \frac{z_0\left(1-e^{(1-\pi)t}\right)}{1-\pi}\right)\right)\right)\right)\delta\pi + O\left(\delta\pi^2\right), \tag{A.7}$$

where $\hat{z}_t = z_0 / \left(z_0 + (1-z_0)e^{-(1-\pi)t}\right)$ is the total population size at time t for the neutral case ($\delta\pi = 0$). Darwinian fitness (w) is given by the number of persister cells in the focal lineage at time T, i.e. $w = P_T = P_t|_{t=T}$.

We now perform an evolutionary stability analysis, and ask: which resident strategy π (performed in the focal population by the lineage Y) is the best response to itself, so that a rare lineage (the focal lineage X) maximizes its fitness by employing the same strategy π? The ESS persister allocation π^*, when it takes an intermediate value ($0 < \pi^* < 1$), thus satisfies $\mathrm{d}w / \mathrm{d}\delta\pi\,|_{\delta\pi=0,\pi=\pi^*} = 0$ (Taylor 1996), and this yields the exact, implicit solution (1). The condition for an ESS at $\pi^* = 0$ is LHS (1) ≥ 1 when $\pi^* \to 0$, which is never satisfied and so some persister allocation will always be favoured by natural selection. The condition for an ESS at $\pi^* = 1$ is LHS (1) ≤ 1 when $\pi^* \to 1$, which is equivalent to $T \leq 1/(1-z_0)$. In the limit of no resource competition ($z_0 \to 0$) this is $T \leq 1$, so here full allocation to persisters ($\pi^* = 1$) is predicted when the time until catastrophe is less than unity, and full allocation to persisters is predicted for increasingly longer waiting times as resource competition increases. In the limit of full resource competition ($z_0 \to 1$) then full allocation to persister function is predicted irrespective of the time until catastrophe.

Other special cases of the parameters yield explicit analytical solutions for π^*. We know that $T \leq 1$ gives $\pi^* = 1$. In the limit of infinite time until catastrophe ($T \to \infty$), (1) obtains $\pi^* = 1/\left(1-(1-p_0)\ln(z_0)\right)$. In the absence of resource competition ($z_0 \to 0$), i.e. exponential growth, then (1) can be solved to yield $\pi^* = 1/T$, and we know that full competition ($z_0 \to 1$) leads to full allocation into persisters ($\pi^* = 1$). Individual cells are motivated to behave for the good of the whole population when the latter is genetically homogenous; here $p_0 = 1$ and $\pi^* = \left(1 + \Omega\left(ze^{T-1}/(1-z)\right)\right)/T$, where Ω denotes the Lambert W-function, or omega function.

Random catastrophes

We now include further realism in our model of persister evolution by relaxing the assumption that catastrophe always strikes at time T; rather, we now consider that it strikes at any time t according to the density function $\phi(t)$. Hence, fitness is given by the expected number of persister cells at the time of catastrophe, i.e. $w = \int_0^\infty \phi(t) P_t \mathrm{d}t$. For illustration, we consider that there is a fixed probability of catastrophe at all times, and hence waiting times are exponentially distributed and we have $\phi(t) = e^{-t/\bar{T}} / \bar{T}$, where $\bar{T}$ is the expected waiting time. This yields an implicit solution for π^*:

$$\int_0^\infty \frac{z_0 p_0 e^{-t/\bar{T}}}{z_0 + (1-z_0)e^{-(1-\pi^*)t}} \left(1 - \pi^* \left((1-p_0)\left(t - \frac{\ln\left(1 - z_0\left(1 - e^{(1-\pi^*)t}\right)\right)}{1-\pi^*} \right) + \frac{p_0(1-z_0)e^{-(1-\pi^*)t}}{z_0 + (1-z_0)e^{-(1-\pi^*)t}} \right) \right) \mathrm{d}t = 0 \quad \text{(A.8)}$$

which can be solved numerically, as illustrated in Figure 3.

Survival, growth and efficiency of resource use

So far we have assumed that persister cells exhibit zero growth and always survive catastrophes, and that nonpersister cells grow and have zero survival through catastrophic events. We have also assumed that persister cells exert the same competitive strain on resources as nonpersister cells. We now extend the basic model to allow persister cells a relative growth rate g and relative survival s, with respect to the growth and survival of nonpersister cells. We also allow bacterial persistence to represent a more efficient use of resurces, with the competitive strain on resources exerted by a persister cell being a fraction a of that exerted by a nonpersister cell. The change in the numbers of cells in lineages X and Y can now be written as:

$$\frac{\mathrm{d}x}{\mathrm{d}t} = \left(1 - (\pi + \delta\pi)(1-g)\right)x_t(1-\zeta_t), \text{ and} \quad \text{(A.9)}$$

$$\frac{\mathrm{d}y}{\mathrm{d}t} = \left(1 - \pi(1-g)\right)y_t(1-\zeta_t), \quad \text{(A.10)}$$

where $\zeta_t = ((\pi+\delta\pi)a+1-(\pi+\delta\pi))x_t + (\pi a + 1-\pi)y_t$ is the 'effective' size of the population, in terms of its total strain on the population's resources, with each persister cell contributing a fraction a of the strain imposed by each nonpersister cell. At carrying capacity we have $\zeta = 1$; the actual number of cells maintained here will depend on the population's allocation to persister function, and is $z = 1$ when no persisters are present. Similarly, we may define an 'effective' frequency $\wp_t = ((\pi+\delta\pi)a+1-(\pi+\delta\pi))x_t/\zeta_t$ of the focal lineage, in terms of its impact on resource competition. We find that the change in these transformed population variables is given by:

$$\frac{\mathrm{d}\zeta}{\mathrm{d}t} = \left(1-(\pi+\wp_t\delta\pi)(1-g)\right)\zeta_t(1-\zeta_t) \text{, and} \tag{A.11}$$

$$\frac{\mathrm{d}\wp}{\mathrm{d}t} = -\wp_t(1-\wp_t)(1-g)(1-\zeta_t)\delta\pi . \tag{A.12}$$

Using the same procedure as outlined previously, we obtain the general solutions:

$$\zeta_t = \frac{\zeta_0}{\zeta_0+(1-\zeta_0)e^{-(1-\pi(1-g))T}}\left(1-\wp_0\left(1-\frac{\zeta_0}{\zeta_0+(1-\zeta_0)e^{-(1-\pi(1-g))T}}\right)T(1-g)\delta\pi\right)+O\left(\delta\pi^2\right), \tag{A.13}$$

$$\wp_t = \wp_0 - \wp_0(1-\wp_0)\left(T-\frac{\ln\left(1-\left(e^{(1-\pi(1-g))T}\right)\zeta_0\right)}{1-\pi(1-g)}\right)(1-g)\delta\pi + O\left(\delta\pi^2\right). \tag{A.14}$$

These can be back-transformed to give z_t and p_t in terms of z_0, p_0 and the other model parameters. Again, we assume that Darwinian fitness is proportional to the number of focal lineage cells that survive the catastrophe at time T:

$$w = p_T z_T\left((\pi+\delta\pi)+\left(1-(\pi+\delta\pi)\right)s\right). \tag{A.15}$$

Substituting in our expressions for z_t and p_t, and evaluating at $t = T$, we then employ the usual procedure to find the condition for (internal) ESS, which is:

$$\frac{\pi^* + (1-\pi^*)s}{1-s}\left[p_0 \frac{\left(1-e^{(1-\pi^*(1-g))T}\right)(1-a)z_0 + \left(1-(1-\pi^*(1-a))z_0\right)(1-g)T}{1-\left(1-e^{(1-\pi^*(1-g))T}\right)(1-\pi^*(1-a))z_0} + (1-p_0)\left(T - \frac{\ln\left(1-\left(1-e^{(1-\pi^*(1-g))T}\right)(1-\pi^*(1-a))z_0\right)}{1-\pi^*(1-g)}\right)(1-g)\right] = 1. \quad \text{(A.16)}$$

Note that, in the special case of $g = s = 0$, $a = 1$, we recover the basic model and the ESS condition given in equation (1). Illustrative numerical solutions for the extended model are presented in Figure 4.

In all cases, we have neglected de novo mutation, assuming that this is infrequent and introduces only minor variation, as is standard in social evolutionary analyses (Taylor & Frank 1996, Frank 1998). Although mutation can be ignored in higher organisms, it could play a role in microbes, under conditions such long-term infections (West et al. 2006). A model that explicitly incorporated mutational effects would generate genetical variation among clonemates, lowering relatedness, and hence reduce the ESS persister allocation.

References

Frank SA (1998) Foundations of social evolution. Princeton: Princeton University Press.

Taylor PD (1996) Inclusive fitness arguments in genetic models of behaviour. J. Math. Biol. 34: 654-674.

Taylor PD, Frank SA (1996) How to make a kin selection model. J. Theor. Biol. 180: 27-37.

West SA, Griffin AS, Gardner A, Diggle SP (2006) Social evolution theory for microorganisms. Nature Rev. Microbiol. 4: 597-607.
